# Examining Adult Protective Services Outcomes: Services Associated With the Decrease of Mistreatment Differed by Elder Mistreatment Type

**DOI:** 10.1093/geront/gnac040

**Published:** 2022-03-22

**Authors:** Pi-Ju Liu, Zachary Hass, Sara K Stratton, Karen M Conrad, Kendon J Conrad

**Affiliations:** School of Nursing and Center on Aging and the Life Course, Purdue University, West Lafayette, Indiana, USA; Schools of Nursing and Industrial Engineering and Regenstrief Center for Healthcare Engineering, Purdue University, West Lafayette, Indiana,USA; San Francisco Adult Protective Services, Department of Aging and Adult Services, City and County of San Francisco, San Francisco, California, USA; Division of Health Policy and Administration, School of Public Health, University of Illinois at Chicago, Chicago, Illinois, USA; Division of Health Policy and Administration, School of Public Health, University of Illinois at Chicago, Chicago, Illinois, USA

**Keywords:** Elder abuse, neglect, and exploitation, Identification, Services, and Outcomes (ISO) Matrix, Standardization

## Abstract

**Background and Objectives:**

Adult Protective Services (APS) are the frontline agencies investigating elder mistreatment and providing/coordinating postinvestigation services. Yet, their effectiveness in reducing different types of mistreatment in relation to services is unknown. This study aimed to address the knowledge gap by identifying services provided by mistreatment type, and examining the associations of services with mistreatment reduction.

**Research Design and Methods:**

A pretest–post-test design was implemented using the Identification, Services, and Outcomes (ISO) Matrix to assess mistreatment levels during case investigation and at case closure after services were provided. San Francisco and Napa APS participated in a 6-month data collection.

**Results:**

The 4 most prevalent types of mistreatment were examined: emotional, physical, financial abuse, and neglect by others. On average, level of mistreatment decreased across mistreatment types after APS intervention. Care/case management, mental health, and other services were most common, while specific services differed depending on type of mistreatment. Care/case management services were associated with physical and emotional abuse reduction, legal services further correlated with emotional abuse reduction; financial planning services were associated with financial abuse reduction; care/case management and other services were associated with neglect reduction.

**Discussion and Implications:**

This is the first study to address APS services by mistreatment type and the outcomes of services. Adoption of the ISO Matrix by APS programs opens the possibility of research and practice collaboration in APS outcomes research using a standardized approach.

Adult Protective Services (APS) are the frontline agencies responding to elder mistreatment reports. As awareness of elder mistreatment grows and the population ages, reports accepted for investigation increased 15.2% from 2016 to 2018 nationwide (Aurelien et al., 2019). The agencies are dedicated to investigating allegations of mistreatment, and decisions are categorized as confirmed, inconclusive, or unfounded. Thirty-three percent of APS clients were identified as victims after APS investigations (McGee & Urban, 2021). APS agencies also assess clients’ needs, make service recommendations, work with clients to obtain agreement on a service plan, and implement the service plan through advocacy, referral, or provision of services (Liu & Anetzberger, 2019). Although APS agencies exist in every state and territory, their infrastructures differ as the lack of federal policy and appropriations have resulted in variations in program structure (Liu & Delagrammatikas, 2021). For example, substantiation rates differed among California county APS programs due to differing interpretations of decision categories (Mosqueda et al., 2016). Ideally, APS should monitor the status of services and not close a case until the victim is safe (Aurelien et al., 2019). In reality, postinvestigation services also differ, such that some APS close clients’ cases after referring clients to other service providers, while others provide more extensive follow-ups. Unfortunately, there has been a dearth of research on the documentation of services and their effectiveness.

In the updated national voluntary consensus guidelines for state APS programs (Administration for Community Living, 2020), standardized practices were recommended. The overarching objective of our project was to pilot a standardized approach by using the Identification, Services, and Outcomes (ISO) Matrix. The ISO Matrix approach trains APS caseworkers to record mistreatment levels numerically during investigation (identification), document interventions provided to clients (services), and reevaluate mistreatment levels again at case closure (outcomes). Given the accomplishment of this objective (Conrad et al., 2021; Liu et al., 2020), this study addressed the following two objectives: (1) to identify kinds and quantities of services provided to APS clients by mistreatment type using the standardized assessment; and (2) to examine the associations of services with mistreatment outcomes. These objectives responded to Jackson’s (2017) call for APS and victim services to determine if services are making a difference in victims’ lives, stating that researchers have “yet to link services to outcomes, let alone unpack the black box that is services” (p. 217). Therefore, our study began examining changes in mistreatment levels and identifying services that ameliorate mistreatment following APS involvement.

## Services Needed by Elder Mistreatment Victims

Studies of services for elder mistreatment victims are rare. The Elder Abuse Prevention Program in New York indicated that 71% of victims received counseling, 64% had services coordinated with professionals, and 63% reported short-term support and advocacy (Dauenhauer et al., 2019). Another study described the interventions for APS clients referred for geriatric assessment in two New Jersey counties, with 46% of victims receiving services from a home health agency, 36% institutional placements, 36% guardianship, 25% urgent medication prescription, and 20% acute hospitalization (Heath et al., 2005). Lastly, of the 40 victims referred to a multidisciplinary team (MDT) in Colorado, services included 76% medical, 73% legal, 47% senior center, and 42% mental health services (Olomi et al., 2019). None of the studies provided details on services by mistreatment type, except the Choi et al. (1999) study focusing on financial abuse. For these cases, 67% of victims received case management, 64% had financial management/representative or protective payee, and some others received legal services.

Services received by victims varied in the above studies, likely because referral sources differed from geriatric assessment to MDT. Based on a report from the National Adult Maltreatment Reporting System (NAMRS), data from eight state APS programs indicated the most common services provided to clients by APS were 14% victim services, 5% care/case management services, and 4% in-home assistance services. In addition, the most common service referrals were 29% other services (such as Alzheimer’s/dementia education, public health, burial/cremation, animal control, and consultation), 6% care/case management services, and 4% medical and dental services (Aurelien et al., 2018). These numbers were much lower than other referral sources, because all alleged cases and types of abuse were included in the NAMRS rather than only confirmed mistreatment cases.

## Theoretical Framework and Measurement Considerations in APS Outcomes Studies

In addition to identifying services provided to victims of each mistreatment type, another need in APS research is to define outcomes associated with services. Researchers reported the dearth of information when examining outcomes in APS cases (Ernst et al., 2014), as well as the lack of appropriate outcomes measured (Burnes et al., 2021). APS outcomes studies have not focused on services, and various outcomes were used, including case closure reasons (Goodrich, 1997), investigation decisions (Payne & Gainey, 2005), changes in mistreatment since APS involvement (Jackson & Hafemeister, 2012; Roberto & Teaster, 2005; Roberto et al., 2007; Wangmo et al., 2014).

Burnes (2017) developed a conceptual practice model to describe community elder mistreatment interventions. The three practice model orientations include harm-reduction, client-centered, and multidisciplinary. APS agencies aim to reduce mistreatment (i.e., harm-reduction) by designing a service plan tailored to each victim’s goals and needs (i.e., client-centered), and are responsible for providing or making referrals for various services (i.e., multidisciplinary) accepted by victims. Burnes differentiated potential intervention results into short-term (client’s engagement), intermediate (service acceptance/refusal), and long-term outcomes (mistreatment alleviation). The mission of APS agencies is to reduce mistreatment to keep clients safe. Therefore, following this conceptual practice model, we were most interested in the long-term outcomes of APS involvement to account for the fact that in addition to investigation of mistreatment, postinvestigation services are part of APS responsibilities.

A major consideration in outcomes measures is how to quantify outcomes. Previous studies adopted binary outcomes (i.e., positive or negative closure reasons, unfounded or confirmed substantiation, risk of mistreatment reduced or not, at-risk or safe from future mistreatment) that were based on raters’ subjective judgment. Burnes, Lachs, et al. (2018) argued against the binary approach, and promoted a severity framework capturing changes of elder mistreatment resulting from the intervention. However, a major barrier limiting APS outcomes research is the lack of standardized outcomes measures (Burnes, Connolly, et al., 2018; Burnes et al., 2021). Measures used by professionals, including APS caseworkers, were found to lack evidence in detecting mistreatment or could not be used directly with victims (Ayalon et al., 2016).

To capture long-term outcomes defined by Burnes (2017), we developed the ISO Matrix to measure mistreatment levels during case investigation and at case closure, as well as services provided to clients by mistreatment type. Because it was not practical to randomly assign APS clients to a no-service control group, a one-group pretest–intervention–post-test design with all clients visited face-to-face by APS caseworkers was chosen. A key to this ISO Matrix as an advance in research is that it employed empirically validated and standardized measures of elder mistreatment from the Elder Abuse Decision Support System (EADSS; see details in Method section). Given the successful implementation of the ISO Matrix, which was the overarching project objective as reported in Conrad et al. (2021) and Liu et al. (2020), the objectives of the current study were as follows:

Objective 1. To identify kinds and quantities of services provided to APS client by mistreatment type.
Objective 2. To examine the associations of services with mistreatment outcomes.

Because APS’ mission is to promote client safety, we hypothesized that on average, post-test score would be lower than pretest regardless of mistreatment type. Although each victim might have various goals and needs, we hypothesized that some services might be more common across mistreatment types, while others would be specific to address one type of mistreatment. No previous studies examined services and their relationship with mistreatment reduction by type, so findings would provide some indication of service effectiveness.

## Method

### Participants and Procedures

San Francisco and Napa APS caseworkers (*n* = 37) and supervisors (*n* = 8) in California participated in a pilot to use the ISO Matrix over 6 months between August 2018 and January 2019. San Francisco APS serves the diverse populations living in urban and suburban areas, while Napa covers suburban and rural environments. Caseworkers and supervisors attended a daylong training offered by the research team covering varied definitions of APS outcomes, outcomes used in the ISO Matrix, the use of the ISO Matrix in the field, and ISO Matrix documentation in their counties’ case management system called LEAPS. During the training, the research team explained that the ISO Matrix aims to improve practice consistency, and that data would not be used for individual caseworker’s performance review. Even though the ISO Matrix assessments are framed in a way that can be asked of clients directly (see Supplementary Material A for indicators of the four most prevalent mistreatment types with an alleged abuser: emotional, physical, financial abuse, and neglect by others), all answers on the ISO Matrix are based on the investigation, including caseworker’s observation, interview with client, alleged perpetrator(s), collaterals, and other supporting evidence.

Across the 6-month period, the ISO Matrix was used as part of APS practice to capture mistreatment levels before and after APS services. Between the two California counties, 556 elder mistreatment cases were investigated face-to-face (see Table 1), with at least one of the four most prevalent mistreatment allegations. Sample sizes for the four mistreatment types ranged from 144 to 281 (see Table 2) with the total number of 832, because some cases involved more than one type of mistreatment. Self-neglect was excluded from analysis, because those cases did not have an alleged abuser. Sample sizes for the other mistreatment types and polyvictimization were too small to conduct inferential statistics, and were excluded from the study.

**Table 1.** Descriptive Statistics of APS Case Demographics (*N* = 556)

Variable	Mean (range) or %	Valid cases
Age	78 (65–105)	556
Female	59%	541
Race		
White	38%	492
Asian	27%	492
Black	18%	492
Hispanic	14%	492
Other	3%	492
Primary language		
English	65%	536
Asian languages	21%	536
Spanish	9%	536
Other non-English	5%	536
Speaks English	75%	556
Marital status		
Single	31%	364
Widowed	31%	364
Married or partnered	30%	364
Divorced or separated	8%	364
Living arrangement		
With others	34%	507
Lives alone	30%	507
With alleged abuser	21%	507
Other	15%	507
In-Home Supportive Services status before APS services	24%	556
Able to give consent to services	87%	540
Alleged abuser		
Family member	67%	333
Nonfamily members known to client	15%	333
Other	10%	333
Caregiver	8%	333

*Notes*: APS = Adult Protective Services. Denominator for percentages is the number of valid cases.

**Table 2.** Counts of Services Assigned to Address Specific Mistreatment Type for Adults 65+

Service type	Emotional abuse (*n* = 281)		Physical abuse (*n* = 144)		Financial abuse (*n* = 253)		Neglect by others (*n* = 154)	
	Advocacy	Direct	Advocacy	Direct	Advocacy	Direct	Advocacy	Direct
Care/case management services	23 (8%)	5 (2%)	16 (11%)	5 (4%)	19 (8%)	8 (3%)	15 (10%)	5 (3%)
Emergency assistance and material aid services	0 (0%)	6 (2%)	0 (0%)	10 (7%)	0 (0%)	5 (2%)	0 (0%)	4 (3%)
Financial planning services	3 (1%)	3 (1%)	0 (0%)	0 (0%)	14 (6%)	4 (2%)	0 (0%)	0 (0%)
Housing and relocation services	9 (3%)	0 (0%)	3 (2%)	0 (0%)	5 (2%)	0 (0%)	5 (3%)	0 (0%)
In-home assistance services	6 (2%)	0 (0%)	4 (3%)	0 (0%)	6 (3%)	0 (0%)	11 (7%)	0 (0%)
Legal services	17 (6%)	0 (0%)	12 (8%)	0 (0%)	6 (2%)	3 (1%)	2 (1%)	0 (0%)
Medical and dental services	11 (4%)	1 (0%)	3 (2%)	1 (1%)	3 (1%)	1 (0%)	13 (8%)	3 (2%)
Mental health services	0 (0%)	79 (28%)	0 (0%)	47 (33%)	0 (0%)	51 (20%)	0 (0%)	30 (20%)
Public assistance benefits	2 (1%)	3 (1%)	5 (3%)	1 (1%)	3 (1%)	1 (0%)	2 (1%)	1 (1%)
Transportation	0 (0%)	1 (0%)	0 (0%)	1 (1%)	0 (0%)	0 (0%)	0 (0%)	2 (1%)
Victim services	14 (5%)	19 (7%)	14 (10%)	20 (14%)	12 (5%)	17 (7%)	3 (2%)	5 (3%)
Other services	15 (5%)	83 (30%)	9 (6%)	41 (28%)	12 (5%)	60 (24%)	4 (3%)	45 (29%)

*Notes*: Denominator for percentages is the number of cases by the type of abuse. Sum of percentages for each type of abuse is larger than 100%, because a client could receive services in different categories.

All data were entered into San Francisco and Napa APS’ case management system, and deidentified data were transferred from the system to the research team for data cleaning and coding before analyses. No consent was obtained because the assessments were incorporated as part of APS practice and data shared with the research team were deidentified. Institutional Review Board of Purdue University (IRB Protocol # 1812021397) deferred the approval to University of California, San Francisco (IRB # 17-23904) to provide annual oversight.

### Measures

The ISO Matrix has three sections: I stands for the identification of mistreatment, S stands for services provided to address mistreatment, and O stands for outcomes.

The identification of mistreatment is based on the EADSS, developed by Conrad and Iris (2015). It is a theory-based system developed through literature review, concept mapping, and testing in the field (Conrad et al., 2017, 2019; Conrad, Iris, Ridings, Langley, et al., 2010; Conrad, Iris, Ridings, Langley, et al., 2010). The EADSS includes comprehensive and structured interview guides to assess the four types of elder mistreatment. Because the full-length EADSS was judged to be too burdensome for caseworkers in the field, short forms were derived (Beach et al., 2017; Conrad et al., 2021) and used for this study. These assessments were administered to capture mistreatment levels during case investigation, and called “pretest” in the counties’ case management system. Any indicator answered as *yes* was scored as 2, *some indication* was scored as 1, and *no* was scored as 0 (see Supplementary Material A). Caseworkers also had the choice for *don’t know* (did not get this information) and *refused* to be answered by client, which were coded as missing data. The items were summed, respectively, by type of mistreatment to allow a quantification of mistreatment level. Because numbers of indicators differed by mistreatment type, possible points for physical abuse ranged from 0 to 6 (3 items), neglect by others from 0 to 14 (7 items), emotional and financial abuse from 0 to 22 (11 items).

Services were documented in the counties’ case management system under client’s service plan. Caseworkers selected the service offered to clients from their county’s list of services, then designated the type(s) of mistreatment the service aimed to address (e.g., pressure ulcer treatment/education aims to resolve neglect by others), and whether the client accepted the service. APS programs do not offer a standard list of services, so while each county offers many services, similar services might have different labels. Because the two counties’ service lists were not identical, the research team worked with the counties’ APS leadership to map all of the services to the 18 service categories established by NAMRS (Aurelien et al., 2019; see Supplementary Material B). Additionally, the NAMRS service categories were divided into advocacy and direct services. Advocacy was done by APS caseworkers, such as advocating with a utility company or landlord to accept a payment plan from a client to avoid utility shutoff or eviction. On the other hand, direct services are either services provided by caseworkers themselves or referrals for direct services made by caseworkers to other agencies, such as home-delivered meals by Meals on Wheels. Because referrals to other agencies had not always materialized by case closure when the assessments were conducted again, two experts (retired San Francisco APS supervisor and manager) independently reviewed the service list to identify the services always delivered by case closure. When the experts did not agree, the research team consulted with each county’s leadership for the final designation (see Supplementary Material B). Only available services accepted by clients that were delivered by case closure were included in this study, because one of our objectives was to examine services that were associated with reduced mistreatment levels, that is, outcomes, and the outcomes were obtained at case closure.

Outcomes, called “post-tests” in the counties’ case management system, were the readministration of the EADSS short forms at case closure. The measurement strategy was to assess the mistreatment level at case closure by type of mistreatment using the same validated measures as during case investigation.

### Data Analysis

The pretest–post-test design allowed for assessment of mistreatment levels before and after service delivery. For each type of mistreatment, only those cases that had a nonzero entry for either the pretest or post-test were included. This means pretest could be zero but not post-test (i.e., mistreatment was found at case closure but not during investigation), or post-test could be zero but not pretest (i.e., indication of mistreatment disappeared after APS intervention).

Our interest was in understanding which services were associated with a reduction in mistreatment by type, so primary independent variables were the services delivered to clients. In order to estimate the relationship between the NAMRS service categories and the change in mistreatment, we utilized the regressor variable and change score models of linear regression discussed and compared by Allison (1990). Model 1, the regressor variable model, included the pretest score and NAMRS service categories as independent variables, and post-test as the dependent variable. Model 2, the change score model, used the change score of post-test minus pretest as the dependent variable and included the NAMRS service categories as independent variables. Both models are useful in the quasiexperimental design setting of pre- and posttreatment measurement of outcomes. The choice between the two models hinges largely upon the degree to which the pretest score determines treatment (i.e., service offerings) and is not straightforward in this case. Therefore, both models are presented, and triangulation of the results is used for drawing inference.

Each model included the services that were assigned for a mistreatment type in a sufficient number of observations (minimum 5% of cases) to prevent unusual cases from driving the results. Assumptions of linear regression were checked using the residuals. False discovery rate (FDR) was controlled at 5% using the Benjamini–Hochberg procedure (1995), which means the expected proportion of rejected null hypotheses that are false positives is 5%. The strongest evidence exists for those services that were statistically significant when controlling for the FDR, and the additional services that were statistically significant prior to FDR control indicate a weaker form of evidence that is worth monitoring.

## Results

Clients’ average age was 78 years old and 59% were female. Their backgrounds were diverse: only 38% of clients were White, and 35% of clients’ primary language was not English. Twenty-four percent of clients were receiving services from the In-Home Supportive Services program when they met with APS caseworker, which means they were Medicaid beneficiaries in California. Sixty-seven percent of the alleged abusers were family members, 15% were nonfamily members, 10% were caregivers, and 8% other (see Table 1 for complete demographics).

### Objective 1: Identify Services Provided by Mistreatment Type

Out of the 18 NAMRS service categories, “caregiver support,” “community day services,” “education, employment, and training,” “medical rehabilitation,” “nutrition,” and “substance use services” were not delivered to anyone at case closure, and were therefore excluded. Table 2 displays the 12 service categories provided either through advocacy or direct service. Across all types of mistreatment, care/case management, mental health, and other services were provided to over 5% of clients both through advocacy or directly. Around one third of services were categorized as “other,” which included language assistance/translation, resources provided to alleged abusers, and some service items labeled as other. Additionally, emotionally abused clients received legal services (6%) and victim services (12%); financially abused clients received financial planning services (8%) and victim services (12%); physically abused clients received emergency assistance (7%), legal services (8%), and victim services (24%); neglected clients received in-home assistance (7%) and medical/dental services (10%).

### Objective 2: Examine the Associations of Services With Mistreatment Outcomes

Table 3 displays the modeling results for the four mistreatment types. Assumptions of linear regression were adequately met for all models. In Model 1, pretest and services provided to over 5% of cases (either advocacy or direct services) were predictors of post-test. In Model 2, services were predictors of the change score on post-test minus pretest. Statistically significant pretest coefficients that were less than one in Model 1 indicated a general reduction in mistreatment across the four mistreatment types. This effect was a 72% decrease for neglect by others, 62% decrease for physical abuse, 43% decrease for emotional abuse, and 31% decrease for financial abuse. Compared with Model 2, Model 1’s results tend to be more conservative.

**Table 3.** Regression Models of Effective Services by Mistreatment Type for Adults 65+

Model		Emotional abuse (*n* = 281)		Physical abuse (*n* = 144)		Financial abuse (*n* = 253)		Neglect by others (*n* = 154)	
		1	2	1	2	1	2	1	2
Pretest		0.57^#^	—	0.38^#^	—	0.69^#^	—	0.28^#^	—
Care/case management services	Advocacy	1.34	−0.30	−0.38	−0.95	0.55	0.41	−0.58	−1.85^#^
	Direct	−3.21*	−4.04*	−2.57^#^	−3.23^#^	−1.54	−1.00	−0.24	1.01
Emergency assistance/ material aid services	Advocacy	—	—	—	—	—	—	—	—
	Direct	—	—	−0.55	−0.41	—	—	—	—
Financial planning services	Advocacy	—	—	—	—	−2.35*	−3.55^#^	—	—
	Direct	—	—	—	—	−3.04	−4.80*	—	—
In-home assistance services	Advocacy	—	—	—	—	—	—	−0.05	−0.29
	Direct	—	—	—	—	—	—	—	—
Legal services	Advocacy	−1.27	−2.01*	0.54	−0.36	—	—	—	—
	Direct	—	—	—	—	—	—	—	—
Medical and dental services	Advocacy	−0.89	−0.78	—	—	—	—	0.21	−0.23
	Direct	−2.59	0.63	—	—	—	—	−1.41	−2.30
Mental health services	Advocacy	—	—	—	—	—	—	—	—
	Direct	0.68	0.11	−0.11	−0.38	−0.54	−0.57	−0.12	0.16
Victim services	Advocacy	−0.89	−1.50	0.85	0.23	2.07	1.16	−1.40	−1.43
	Direct	1.29	0.87	0.31	0.13	0.79	0.19	−0.04	−1.71
Other services	Advocacy	−0.56	−0.32	−0.79	−0.58	−0.95	−1.02	−0.10	−0.38
	Direct	−0.11	−0.52	0.50	0.16	−0.17	−0.41	−0.26	−1.45^#^
Intercept		0.22	−1.80	0.77	−0.57	−0.01	−1.49	0.76	−0.89
*R* ^2^		0.40	0.06	0.21	0.11	0.42	0.08	0.12	0.18
Total score range		0–22		0–6		0–22		0–14	

*Notes*: —: Less than 5% of cases of the mistreatment type received that service, so it was not included in the model. Model 1 dependent variable was post-test. Model 2 dependent variable was the change from pretest to post-test, using scores of post-test minus pretest. A negative service coefficient or pretest less than one implies the variable is correlated with mistreatment reduction.

*p < .05. ^#^Statistically significant when controlling false discovery rate at 5%.

#### Emotional abuse

The provision of direct care/case management services was associated with reduced mistreatment levels by an average of 3.2 points in Model 1 (*p* = .035, 15% reduction from Maximum Score Possible [MSP]) and 4 points in Model 2 (*p* = .019, 18% MSP reduction). For context, all items on each scale were worth 2 points if marked as indicated, 1 if marked as some indication, and 0 otherwise. Advocacy of legal services further reduced mistreatment levels by 2 points in Model 2 (*p* = .035, 9% MSP reduction).

#### Physical abuse

Direct provision of care/case management services was strongly associated with a 2.6 drop in post-test scores in Model 1 (*p* = .004, 43% MSP reduction), and a 3.2 drop from pretest to post-test scores in Model 2 (*p* = .001, 54% MSP reduction).

#### Financial abuse

The advocacy for financial planning services was associated with reduced post-test scores by 2.4 points in Model 1 (*p* = .025, 11% MSP reduction) and with reduced change scores by 3.6 points in Model 2 (*p* = .001, 16% MSP reduction). Direct provision of financial planning services was associated with an average reduction of 4.8 points in Model 2 (*p* = .015, 22% MSP reduction).

#### Neglect by others

No service provision or advocacy was strongly associated with post-test scores in Model 1, while advocacy for care/case management services and other direct services was associated with reduced mistreatment levels by an average of 1.9 (*p* = .010, 13% MSP reduction) and 1.5 points (*p* = .002, 10% MSP reduction) in Model 2, respectively.

## Discussion

This study used the ISO Matrix as a standardized approach to (1) identify services provided to APS client by mistreatment type, and (2) examine services that were associated with mistreatment reduction by type. The four most prevalent types were examined: emotional, financial, physical abuse, and neglect by others. A general reduction in mistreatment was observed across all types, demonstrated by the significant pretest predictor in Model 1. It is important to note that APS’ mission is to ensure client’s safety, which can often be achieved without eradicating mistreatment entirely but by reducing it. Client’s self-determination, for those with decision-making capacity, is respected in APS work (Burnes, 2017; Liu & Anetzberger, 2019). For example, if an abuser continues to stay in the client’s life and the client does not want the abuser to be removed, protective gatekeeping actions might be put in place, but mistreatment may still occur as the abuser maintains access to the client.

Services that were delivered by the time of case closure were included in this study. As hypothesized, some services were more common across mistreatment types, including care/case management, mental health, and other services. Victim services were more likely to be received by all victims except for those neglected by others, while legal services were more likely to be received by emotional and physical abuse victims. Physical abuse victims were also more likely to receive emergency assistance, and financial abuse victims were likely to receive financial planning services. Victims of neglect were more likely to receive in-home assistance and medical and dental services.

About one third of the clients received “other services,” which was at least partially a function of the diverse population living in San Francisco and Napa. A quarter of clients did not speak English and one third spoke English as a second language, indicating a high need for translation services. However, translation was not one of the services listed on NAMRS service categories, so it was included in the “other” category. Another major service in this category was services provided to alleged abusers. Because elder mistreatment victims oftentimes cannot or prefer not to be separated from their abusers, especially when abusers are family, understanding services offered to the alleged abuser could facilitate alternative mistreatment resolution (Moore & Browne, 2017; Penhale, 2010).

As presented in Burnes’ (2017) conceptual model, long-term outcomes were estimated to understand the relationship of particular services with reduced mistreatment by type. Effective services differed by mistreatment type. The strongest evidence was that direct care/case management was associated with physical abuse reduction, likely because care/case managers served as a gatekeeper to check in regularly. Care/case management, along with legal services, was associated with emotional abuse reduction. Evidence of emotional abuse is hard to detect, but the nonphysical harm is sometimes more painful and has long-lasting effects (Seff et al., 2008). Because abusers are often angry and cannot control their temper (Liu et al., 2019), legal interventions might be necessary to help remove victims from emotional devastation. Financial abuse victims who received some form of financial planning service or advocacy were in a better position at case closure (Lichtenberg et al., 2019). Lastly, advocacy of care/case management and direct “other services” were associated with neglect reduction. It is unclear which “other services” drove the significant results.

### Practice Implications

A key contribution of this study was the systematic approach in measuring mistreatment levels, documenting APS services, and examining services’ impact on reducing mistreatment. This standardized approach in implementing the ISO Matrix informed effective interventions that lead to clients’ safety. Although a single study in one geographic area did not have the power to draw widely generalizable conclusions, participating APS programs were impressed that their efforts could be measured quantitatively with their practice standardized. The indicators in the ISO Matrix served as prompts for caseworkers to conduct investigations, and a way to converse with clients to build rapport. As a result, the two counties decided to formally adopt the ISO Matrix into their practice, supporting proof of concept of the ISO Matrix. It also fostered expectations that standardized data can provide guidance that was not available when structured protocols are lacking (Liu et al., 2020). Lastly, using the NAMRS to categorize services promotes consistency in service documentation across jurisdictions. Perhaps a major accomplishment of the study was that both counties have continued using the ISO Matrix after the pilot, and as additional counties and states adopt this systematic approach, using the ISO Matrix and NAMRS categories will allow future data to be collected and analyzed across jurisdictions.

### Limitations and Future Research

Although our desire was to be able to make a causal interpretation, the effects of service were confounded with time due to the lack of a control group. As a result, our findings should be interpreted as correlational rather than causal. Nevertheless, even the correlational information was liable to be useful both in service provision assessment and the design of future interventions and research. Although setting up a control group of no service is not ethical, future research could consider comparison groups without service, for example, APS clients who refused services, or clients who cannot receive services due to lack of service providers. Secondly, the service categories with less than 5% of cases were omitted from the regression models. The small samples did not provide for adequate estimation of effects and may represent atypical cases that would unduly affect inference. We were also not able to account for other program services provided to clients that APS caseworkers were not aware of, nor some service referrals on the waitlist to be executed. Relationship between services and outcomes among less common mistreatment types and the complex phenomenon of polyvictimization could not be examined due to the small sample and missing data.

Although the study covered clients of diverse backgrounds, it represented only two counties in a single state, so replication of this approach is needed in other settings. As the data on services and outcomes accumulate, results can be tested with greater sample sizes and varying populations, allowing a firmer verdict on causality to be rendered. Moreover, the same APS caseworker conducted the pre- and post-tests and connected the client to services. It was impractical to have post-test conducted by another caseworker. This was not only due to financial and workload considerations, but best practices encouraged having the same caseworker develop rapport with a client to achieve client-centered interventions. Nonetheless, future research might consider ways to have a third party complete the post-test for objectivity. Lastly, we informed the caseworkers to document levels of mistreatment at case closure without projecting what would happen in the future. Future research could conduct follow-up to assess longer-term outcomes beyond case closure, and undertake comparative evaluation of services to expand the long-term outcomes defined, such as health or quality of life.

## Conclusion

APS agencies have lacked robust evidence and high-quality studies to identify effective services by mistreatment type. This study began to address this critical need to narrow the knowledge gap in the field. Integrating meaningful data collection in practice would help APS agencies verify whether or not their work was successful. If successful, data would reflect how APS agencies achieved success, so that best practices could be repeated. If not, data would show how practice could be improved. Although quite basic at this time, services’ correlation with hypothesized outcomes using the calculation of post-test and pretest scores provided some indication of effectiveness that could be further examined in future replications.

## Supplementary Material

gnac040_suppl_Supplementary_Material_S1

gnac040_suppl_Supplementary_Material_S2
