# Supplementary material for: Examining Adult Protective Services Outcomes: Services Associated With the Decrease of Mistreatment Differed by Elder Mistreatment Type
Source: Gerontologist. 2022 Mar 22;62(9):1359–68. doi: 10.1093/geront/gnac040 (PMC9579456; doi:10.1093/geront/gnac040)
Supplement: gnac040_suppl_Supplementary_Material_S1 [file gnac040_suppl_supplementary_material_s1.docx]

**Online Supplementary Material** **A. The Identification, Services, and Outcomes Matrix Items Used in Pretest and Posttest: Emotional Abuse, Financial Abuse, Physical Abuse, and Neglect by Others**

*Emotional abuse* is the infliction of fear, anguish, agitation, or other emotional distress through verbal or nonverbal acts.

| **Directions: Please check a box after each question *(all questions refer to past 12 months, including the present).***  **Yes** – means that the problem is directly observable or reported by client or collateral; **Some indication** – means suspicion that the problem is there, but there is no unequivocal proof; **No** – means there is no evidence or suspicion that abuse is occurring; **DK** - means there is no information available to make the decision. **RF –** the client or collateral refused to answer. | **Yes (2)** | **Some Indication (1)** | **No (0)** | **Don’t Know (.)** | **Refused (.)** |
| --- | --- | --- | --- | --- | --- |
| **EMO1-1.** Has ____ **manipulated** or **tried to control** you? |  |  |  |  |  |
| **EMO2-1.** Have you been **uncomfortable** with ____? |  |  |  |  |  |
| **EMO3-1.** Has ____ called you **unkind names** or **put you down**? |  |  |  |  |  |
| **EMO4-1.** Has ____ **kept things from you** or **lied** about things you should know about? |  |  |  |  |  |
| **EMO5-1.** Has ____ **used nonverbal behavior** such as shaking a fist or other threatening gestures? |  |  |  |  |  |
| **EMO6-1.** Have you ever been **afraid** of ____? |  |  |  |  |  |
| **EMO7-1.** Has ____ **failed to support** or **back you up** when you needed it? |  |  |  |  |  |
| **EMO8-1.** Has ____ **made you feel small**, such as treating you as a child? |  |  |  |  |  |
| **EMO9-1.** Has ____ **talked about you as if you were not there**? |  |  |  |  |  |
| **EMO10-1.** Has ____ **not let you speak** for yourself? |  |  |  |  |  |
| **EMO11-1.** **Other indicator(s) of emotional abuse.** |  |  |  |  |  |
| **EMO12-1.** Please feel free to provide additional information, if you answered “**Yes**” or “**Some Indication**” to any of the questions above.  ____________________________________________________________________________________________________________________________________________________________________________________________________________ |  |  |  |  |  |

*Financial abuse* is the illegal or improper use of a client’s funds, property or assets.

Note: “Undue influence” means excessive persuasion that causes another person to act or refrain from acting by overcoming that person’s free will and results in inequity.

| **Directions: Please check a box after each question *(all questions refer to past 12 months, including the present).***  **Yes** – means that the problem is directly observable or reported by client or collateral; **Some indication** – means suspicion that the problem is there, but there is no unequivocal proof; **No** – means there is no evidence or suspicion that abuse is occurring; **Unknown/NA** - means there is no information available to make the decision, or the question is not applicable. | **Yes (2)** | **Some Indication (1)** | **No (0)** | **Don’t Know (.)** | **Refused (.)** |
| --- | --- | --- | --- | --- | --- |
| **FINA1-1.** Has ____**borrowed** **money** from you but not paid it back? |  |  |  |  |  |
| **FINA2-1.** Has ____**felt entitled** to use your money for him/herself? |  |  |  |  |  |
| **FINA3-1.** Has ____**used your money** on their own behalf instead of for your benefit? |  |  |  |  |  |
| **FINA4-1.** Have there been **unexplained disappearances** of your money or possessions? |  |  |  |  |  |
| **FINA5-1.** Has ____ **lied** about how they were spending your money? |  |  |  |  |  |
| **FINA6-1.** Did ____**take advantage of you** to get a hold of your resources such as a house, car, or money? |  |  |  |  |  |
| **FINA7-1.** Has______ lived with you, but refused to pay their share of expenses? |  |  |  |  |  |
| **FINA8-1.** Has ____**refused to give you an accounting** of how your money was spent? |  |  |  |  |  |
| **FINA9-1.** Have there been **unusual activities** in your bank accounts, for example, large withdrawals, frequent transfers of funds? |  |  |  |  |  |
| **FINA10-1.** Has ____ **promised care for you** but then did not provide it? |  |  |  |  |  |
| **FINA11-1.** **Other indicator(s) of financial exploitation, including fraud and scams.** |  |  |  |  |  |
| **FINA12-1.**  Please feel free to provide additional information, if you answered “**Yes**” or “**Some Indication**” to any of the questions above:  __________________________________________________ |  |  |  |  |  |

*Physical abuse* is the non-accidental use of physical force that results or could have resulted in bodily injury, physical pain, or impairment.

| **Directions: Please check a box after each question *(all questions refer to past 12 months, including the present).***  **Yes** – means that the problem is directly observable or reported by client or collateral; **Some indication** – means suspicion that the problem is there, but there is no unequivocal proof; **No** – means there is no evidence or suspicion that abuse is occurring; **DK** - means there is no information available to make the decision. **RF –** the client or collateral refused to answer. | **Yes (2)** | **Some Indication (1)** | **No (0)** | **Don’t Know (.)** | **Refused (.)** |
| --- | --- | --- | --- | --- | --- |
| **PHYS1-1.** Has ____ attempted or caused you any physical **injuries**? For example, bruises or welts, burns, cuts (external injuries), fractures, or sprains (internal injuries)? |  |  |  |  |  |
| **PHYS2-1.** Have you been a victim of an **attempted or completed physical attack**? For example, hit, kicked, punched, slapped, handled roughly, pushed, shoved, grabbed, shaken, or stabbed by a knife? |  |  |  |  |  |
| **PHYS3-1.** **Other indicator(s) of physical abuse:** For example, being shot at, being tied up, struck by a cane, or something thrown at you? |  |  |  |  |  |
| **PHYS4-1.** Please feel free to provide additional information, if you answered “**Yes**” or “**Some Indication**” to any of the questions above: |  |  |  |  |  |

*Neglect* is defined as the refusal or failure to fulfill any part of a person’s obligations or duties

to a client. Neglect requires there be a person responsible for client’s care. Caseworker should observe whether neglect takes place.

| **Directions: Please check a box after each question *(all questions refer to past 12 months, including the present).***  **Yes** – means that the problem is directly observable or reported by client or collateral; **Some indication** – means suspicion that the problem is there, but there is no unequivocal proof; **No** – means there is no evidence or suspicion that abuse is occurring; **DK** - means there is no information available to make the decision. **RF –** the client or collateral refused to answer. | **Yes (2)** | **Some Indication (1)** | **No (0)** | **Don’t Know (.)** | **Refused (.)** |
| --- | --- | --- | --- | --- | --- |
| **NEGL1-1.** Does the (Alleged Victim) have **poor personal hygiene** as evidenced by a noticeable odor, long and dirty fingernails, etc.? |  |  |  |  |  |
| **NEGL2-1.** Does the (Alleged Victim) **lack needed medications or medical equipment** (including eyeglasses, hearing aids, dentures, walkers, etc.)? |  |  |  |  |  |
| **NEGL3-1.** Does the (Alleged Victim’s) home or yard appear **unsafe or unsanitary** that violates the health and safety codes? For example, having clutter near a heating source, or animal hoarding. |  |  |  |  |  |
| **NEGL4-1.** Are there **odors** in (Alleged Victim’s) home that raise concerns (urine, feces, garbage)? |  |  |  |  |  |
| **NEGL5-1.** Does the (Alleged Victim) show signs of **malnourishment** or **dehydration**? |  |  |  |  |  |
| **NEGL6-1.** Is the (Alleged Victim) left **alone** **without** **adequate supervision**? |  |  |  |  |  |
| **NEGL7-1.** **Other indicator(s) of neglect.** For example, lice, bedbugs, cockroaches, mice and other infestations, dangerous room temperature. |  |  |  |  |  |
| **NEGL8-1.** Please feel free to provide additional information, if you answered “**Yes**” or “**Some Indication**” to any of the questions above: __________________________________________________  __________________________________________________  __________________________________________________  __________________________________________________ |  |  |  |  |  |
