# Supplementary material for: Examining Adult Protective Services Outcomes: Services Associated With the Decrease of Mistreatment Differed by Elder Mistreatment Type
Source: Gerontologist. 2022 Mar 22;62(9):1359–68. doi: 10.1093/geront/gnac040 (PMC9579456; doi:10.1093/geront/gnac040)
Supplement: gnac040_suppl_Supplementary_Material_S2 [file gnac040_suppl_supplementary_material_s2.docx]

**Online Supplementary Material** **B. Service Mapping between San Francisco and Napa Adult Protective Services**

| NAMRS categories | San Francisco service list | | | | Napa service list | | | |
| --- | --- | --- | --- | --- | --- | --- | --- | --- |
|  |  | Expert A | Expert B | Final | Name | Expert A | Expert B | Final |
| Care/Case Management Services | Adv_Community-Based Services | 1 | 1 | 1 | CBO Long-Term Case Management | 0 | ? | 0 |
|  | Adv_Discharge Planners | 1 | 1 | 1 | Referral to Community Agencies | 0 | ? | 0 |
|  | HST_Home Visit Human Service Technician | 1 | 1 | 1 | Referral to MDT | 1 | 1 | 1 |
|  | Ref_Case Management | 0 | 0 | 0 | Referral to Regional Center | 0 | ?/0 | 0 |
|  | Ref_Out-of-County APS Services | 1 | 0 | 0 | Referral to Veterans Services | 0 | ?/0 | 1 |
|  | Ref_Private Conservator | 1 | 0 | 0 | Advocacy with Regional Center (NBRC) | 1 | 1 | 1 |
|  | Ref_Regional Centers/DD Programs | 0 | 0 | 0 | Case Management | ? | ? | 0 |
|  | Ref_Veterans Services | 0 | 0 | 0 | Advocacy with the VA | 1 | 1 | 1 |
|  | Ref_Forensic Center | 1 | 1 | 1 |  |  |  |  |
|  | Svs_Meeting Facilitation/Mediation | 1 | 1 | 1 |  |  |  |  |
| Caregiver Support Services | Ref_Caregiver Services | 0 | 0 | 0 | Referral to Caregiver Services | 0 | 0 | 0 |
| Community Day Services | Ref_ADHC/CBAS Adult Day Health Care/ Community Based Adult Services | 0 | 0 | 0 | Referral to Adult Day Care | 0 | ?/0 | 0 |
| Education, Employment, and Training Services |  |  |  |  |  |  |  |  |
| Emergency Assistance and Material Aid Services | HST_Decluttering Assistance | 1 | 1 | 1 | Emergency Clothing/Medication | 1 | 1 | 1 |
|  | HST_Emergency Medication | 1 | 1 | 1 | Blankets, linens and household goods | 1 | 1 | 1 |
|  | Ref_Bedbug Services | 0 | 0 | 0 | Emergency Shelter | 1 | 1 | 1 |
|  | Ref_SFPD Wellbeing Check | 1 | 1 | 1 | Emergency Response Services | 1 | 1 | 1 |
|  | Svs_Bedbug Eradication | 1 | 1 | 1 | Emergency Food | 1 | 1 | 1 |
|  | Svs_Clothing, Personal & Household Goods | 1 | 1 | 1 | Repair/Replacement of Essential Appliances | 1 | 1 | 1 |
|  | Svs_Deep Cleaning/Debris Removal | 1 | 1 | 1 | Plumbing and Electrical Repair | 1 | 1 | 1 |
|  | Svs_Emergency Food | 1 | 1 | 1 | Law Enforcement Welfare Check | 1 | 1 | 1 |
|  | Svs_Emergency Home Care | 1 | 1 | 1 | Referral to 5150 Evaluation | 1 | 1 | 1 |
|  | Svs_Emergency Response Services | 1 | 1 | 1 | Locksmith | ?/1 | 1 | 1 |
|  | Svs_Emergency Shelter | 1 | 1 | 1 | Referral to Law Enforcement (Welfare Check) | 1 | 1 | 1 |
|  | Svs_Facilitate 5150 evaluation | 1 | 1 | 1 | Heavy Cleaning/Hauling | 1 | 1 | 1 |
|  | Svs_Locksmith | 1 | 1 | 1 | Food | 1 | 1 | 1 |
|  | Svs_Utilities Assistance | 1 | 1 | 1 |  |  |  |  |
|  | Ref_Emergency Shelter | 0 | 0 | 0 |  |  |  |  |
|  | HST_Emergency Food | 1 | 1 | 1 |  |  |  |  |
| Financial Planning Services | Adv_Financial Institutions | 1 | 1 | 1 | Referral for Money Management | 0 | 0 | 0 |
|  | Ref_Money Management | 0 | 0 | 0 | Advocacy with Financial Institutions | 1 | 1 | 1 |
|  |  |  |  |  | Financial Resource Information Provided | 1 | 1 | 1 |
|  |  |  |  |  | Referral for money management (Rep Payee/Fiduciary) | 0 | ?/0 | 0 |
| Housing and Relocation Services | Adv_Homeless Services | 1 | 1 | 1 | Housing Advocacy | 1 | 1 | 1 |
|  | Adv_Landlord/Property Owner | 1 | 1 | 1 | Referral to Homeless Services | 0 | 0 | 0 |
|  | Ref_Homeless Services | 0 | 0 | 0 | Advocacy with Code Enforcement | 1 | 1 | 1 |
|  | Ref_Housing - Advocacy (Non-Eviction) | 0 | 0 | 0 | Referral to Code Enforcement | 0 | 0 | 0 |
|  | Ref_Housing - Eviction Prevention | 1 | 0 | 0 |  |  |  |  |
|  | Svs_Rental Assistance | 1 | 1 | 1 |  |  |  |  |
|  | Ref_Housing Code Enforcement | 0 | 0 | 0 |  |  |  |  |
| In-home Assistance Services | Adv_Home Care/Home Health (non-IHSS) | 1 | 1 | 1 | IHSS Referral | 0 | 0 | 0 |
|  | Adv_IHSS In Home Supportive Services | 1 | 1 | 1 | Referral to Friendly Visitor | 0 | ? | 0 |
|  | Ref_IHSS | 0 | 0 | 0 | Safety Planning | 1 | 1 | 1 |
|  | Ref_Home Care Agencies (non-IHSS) | 0 | 0 | 0 | Recommend Security Devices | 1 | 0 | 0 |
|  |  |  |  |  | Referral to Lifeline | 0 | 0 | 0 |
|  |  |  |  |  | Referral to Home Care Agencies | 0 | 0 | 0 |
| Legal Services | Adv_Legal Providers | 1 | 1 | 1 | Advocacy with Legal Providers | 1 | 1 | 1 |
|  | Ref_Public Guardian | 0 | 0 | 0 | Referral to Public Guardian | ? | ? | 1 |
|  | Ref_Legal Services | 0 | 0 | 0 | Referral to Public Conservator | ? | ? | 1 |
| Medical and Dental Services | Adv_Medical Providers | 1 | 1 | 1 | Advocacy with ER Units | 1 | ?/1 | 1 |
|  | Ref_Medical and Dental Services | 0 | 0 | 0 | Referral to Public Health Nursing | ? | ? | 1 |
|  | Ref_CQA RN Visit Clinical Quality Assurance | 1 | 1 | 1 | Advocacy with Medical Providers | 1 | 1 | 1 |
|  | Svs_Medications & Medical Supplies | 1 | 1 | 1 | Support with Medication Management | ?/1 | ? | 1 |
|  | Ref_Medical Services | 0 | 0 | 0 | Referral to Durable Medical Equipment | 0 | 0 | 0 |
|  | Svs_CQA Nursing Consult Clinical Quality Assurance | 1 | 1 | 1 | Advocacy with Discharge Planners | 1 | 1 | 1 |
| Medical Rehabilitation Services |  |  |  |  |  |  |  |  |
| Mental Health Services | Ref_Mental Health Services | 0 | 0 | 0 | Referral to Counseling | 0 | 0 | 0 |
|  | Svs_Education & Counseling (for client, AA, collaterals) | 1 | 1 | 1 | Referral to Mental Health Services | 0 | 0 | 0 |
| Nutrition | Ref_Home Delivered Meals | 0 | 0 | 0 | Referral to Home Delivered Meals | 0 | 0 | 0 |
| Public Assistance Benefits | Adv_Other City/County/State/Federal Agencies | 1 | 1 | 1 | Adv w/Other City/County/State Federal Agencies | 1 | 1 | 1 |
|  | HST_Benefits Assistance | 1 | 1 | 1 | Benefits Assistance | 0 | ? | 1 |
|  | Ref_Public Benefits (not IHSS or veterans) | 0 | 0 | 0 | Ref. Seasons of Sharing | 0 | 0 | 0 |
|  | Svs_Benefit Assistance | 1 | 1 | 1 | Assistance/Advocacy with Benefits Application | 1 | 1 | 1 |
|  | Ref_Entitlement Application | 0 | 0 | 0 |  |  |  |  |
| Substance Use Services | Ref_Substance Abuse Services | 0 | 0 | 0 | Ref. Substance Abuse Services | 0 | 0 | 0 |
| Transportation | HST_Transport Eviction Defense | 1 | 1 | 1 | Transportation Services | 1 | ? | 0 |
|  | HST_Transport Legal Assistance | 1 | 1 | 1 |  |  |  |  |
|  | HST_Transport Medical Appt. | 1 | 1 | 1 |  |  |  |  |
|  | HST_Transport Other/Misc | 1 | 1 | 1 |  |  |  |  |
|  | Ref_Transportation Services | 0 | 0 | 0 |  |  |  |  |
|  | Svs_Transportation | 1 | 1 | 1 |  |  |  |  |
| Victim Services | Adv_Law Enforcement | 1 | 1 | 1 | Referral to DA Victim/Witness | ? | ? | 0 |
|  | Ref_Domestic Violence Prevention Services | 0 | 0 | 0 | Referral to NEWS | ? | ? | 0 |
|  | Ref_Ombudsman | 0 | 0 | 0 |  |  |  |  |
|  | Svs_SFPD Incident Report | 1 | 1 | 1 |  |  |  |  |
| Other Services | Adv_Other | 1 | 1 | 1 | Collaboration with Client's Support System | 1 | 1 | 1 |
|  | Adv_Utility Companies | 1 | 1 | 1 | General Resource Pamphlet Provided | 1 | 1 | 1 |
|  | HST_Item Pick-Up/Delivery | 1 | 1 | 1 | Resources Provided to AA | 1 | 1 | 1 |
|  | HST_Language Translation | 1 | 1 | 1 | Advocacy with Utility Companies | 1 | 1 | 1 |
|  | Ref_Child Protective Services | 1 | 1 | 1 |  |  |  |  |
|  | Ref_Other | 0 | 0 | 0 |  |  |  |  |
|  | Ref_State Licensing Board | 0 | 0 | 0 |  |  |  |  |
|  | Svs_Obtained Language Assistance | 1 | 1 | 1 |  |  |  |  |
|  | Svs_Other | 0 | 1 | 1 |  |  |  |  |
| *Note.* 0=service usually not delivered by case closure; 1=service usually delivered by case closure; ?=unsure if service delivered by case closure. | | | | | | | |  |
